# Prevalence of housing structure and quality indicators in India: An assessment of changes across 720 districts between 2016 and 2021

**DOI:** 10.1016/j.ssmph.2025.101899

**Published:** 2025-12-14

**Authors:** Anoop Jain, Gary Adamkiewicz, Rockli Kim, S.V. Subramanian

**Affiliations:** a Department of Environmental Health, Boston University School of Public Health, Boston, MA, USA; b Department of Environmental Health, Harvard T.H. Chan School of Public Health, Boston, MA, USA; c Division of Health Policy and Management, College of Health Science, Korea University, Seoul, South Korea; d Interdisciplinary Program in Precision Public Health, Department of Public Health Sciences, Graduate School of Korea University, Seoul, South Korea; e Harvard Center for Population and Development Studies, Cambridge, MA, USA; f Department of Social and Behavioral Sciences, Harvard T. H. Chan School of Public Health, Boston, MA, USA

**Keywords:** Housing quality, Social determinants of health, India, Geographic variation

## Abstract

The extent to which a house is structurally sound is an important marker of housing quality and a determinant of human health. In India, the share of homes that are structurally sound has increased considerably over the past few decades, yet geographical variations persist especially between urban and rural communities. Using data from two rounds of India's National Family Health Survey in 2016 and 2021, we estimated a multilevel model using a Markov Chain Monte Carlo procedure to examine changes in the share of finished, semi-finished, and rudimentary housing in urban and rural communities across India's 720 districts. In urban communities, the share of finished housing increased slightly from 82.9 % (95 % CI: 82.7–83.1) in 2016 to 83.2 % (95 % CI: 83.0–83.4) in 2021. In rural communities, the share of finished housing increased from 41.3 % (95 % CI: 41.1–41.4) in 2016 to 48.5 % (95 % CI: 48.3–48.6) in 2021. However, we found substantial between-district disparities, and that the between-community variation increased in many of the districts that experienced overall improvements in housing quality for all three measures of housing quality between 2016 and 2021. District administrations in India can use these results to understand the quality of housing in their jurisdictions. These results can help district administrators work with national policy makers to refine policies aimed at improving housing quality.

## Highlights

• Access to safe housing is a fundamental human right and a major Sustainable Development Goal.
• However, progress towards safe housing has been unequal across *and* within India's 720 districts.
• Within-district inequality increased in many districts that experienced overall improvements in safe housing.
• We also show that safe housing remains most out of reach of India's rural residents.
• Ensuring access to safe housing is critical in both urban *and* rural communities throughout India.

## 1 Introduction

Housing has long been considered an important social determinant of health (The Lancet, 2024). In 1848, Virchow identified poor housing quality as a root cause of a typhoid epidemic in Upper Silesia (Virchow, 1848). Since then, the right to safe and adequate housing that “supports a state of complete physical, mental, and social well-being” has been enshrined by the United Nations General Assembly (World Health Organization, 2018). And now, the Sustainable Development Goals (SDGs) have set targets to ensure that by 2030, all humans live in settlements that are “inclusive, safe, resilient, and sustainable”. (United Nations Department for Economic and Social Affairs, 2023).

A hallmark of safe and adequate housing is that it is structurally sound. Homes built solely with rudimentary or raw materials, such as mud, straw, cardboard, among others do not typically meet modern structural standards and are thus considered inadequate (Initiative et al., 2019). It should be noted that traditional materials can be used effectively as components of modern design where their physical properties, cost and design aesthetics offer advantages while maintaining structural integrity (Kulshreshtha et al., 2020). But homes built with raw materials can develop cracks in the walls and roof, or they might not be properly insulated, and thereby might not provide protection against pests such as rodents and cockroaches (Bradman et al., 2005). These structural deficiencies can cause exposure to extreme temperatures, dampness and mold, and disease vectors (Hamilton et al., 2011; Jatta et al., 2018; Oreszczyn et al., 2006).

Examining housing quality in India is particularly important given the myriad public health implications. The share of homes built entirely of finished materials increased from 24 % in 1993 to 60 % in 2021 throughout India (International Institute for Population Sciences, 2021; National Family Health Survey, 1992, 1995). However, millions of households are still made with some combination of rudimentary materials such as mud, grass, stones, thatch, straw, unburnt bricks, or leaves (International Institute for Population Sciences, 2021; National Family Health Survey, 1992, 1995). Previous research has shown links between housing quality and adverse health outcomes in India. For example, evidence from Ahmedabad, a city in Gujarat, showed that poor structural quality was associated with adverse physical and mental health outcomes when compared to good structural quality (Vaid, 2023; Vaid & Evans, 2017). In New Delhi, poor structural quality was associated with an increased risk of asthma and acute respiratory infections (Firdaus & Ahmad, 2013). Examining the quality of housing in India is also important given that India's population is rapidly aging (India Ageing Report, 2023). When compared to good housing quality, there was an increased risk of falls among those living in moderate or poor quality homes among older adults across India (Cheng, Shao, et al., 2025). Additionally, evidence from the Longitudinal and Aging Study in India shows that older and aging adults living in homes made from temporary materials were at greater risk of cognitive impairment than those living in homes made from finished materials (Cheng, Wu, et al., 2025). Thus, housing quality in India is a determinant of health outcomes across the life course.

While the share of finished housing has increased throughout India over the past several decades, the extent to which this progress varies between and within India's districts has not been previously studied. Filling this gap is important as it can highlight districts that are lagging in terms of housing quality and could help explain the distribution of health outcomes in these geographies. Evidence shows that the quality of housing varies at much smaller geographic levels, such as within cities (Haque et al., 2020). But districts implement programs such as *Pradhan Mantri Awas Yojana (PMAY),* a nation-wide initiative targeting India's poorest and most marginalized by providing them with financial assistance to build safe and adequate homes with finished walls, roofs, and floors (Chaudhary & Iyer, 2024; Housing for All Mission, 2021). Districts are led by District Magistrates (DM), individuals who oversee program delivery, and ensure that resources being delivered to those who need them the most (Farrington et al., 2002; Sarma, 2020). Therefore, a district's performance towards development goals is inextricably tied to the performance of its administration. Furthermore, DMs have considerable power when deciding which villages or urban wards within the district are prioritized. These administrative factors create social conditions that could help explain within-district between-community variations in health, nutrition, poverty indicators (Jain et al., 2022, 2023; Lee et al., 2022; Rajpal et al., 2021, 2022), and progress towards the Sustainable Development Goals (SDG) (Subramanian et al., 2023). External shocks, such as the COVID-19 pandemic, which disrupted construction material supply chains and caused construction labor shortages, along with the growing threat of flooding due to extreme weather events, could also undermine a district's progress towards improving housing quality (Rani et al., 2022; Saharia et al., 2025). This further underscores the importance of examining housing quality at the district level in India.

Furthermore, SDG 11 calls for universal access to adequate, safe, and affordable housing by 2030 (United Nations Department for Economic and Social Affairs, 2023). However, the indicator for this goal only tracks progress for urban residents (United Nations Department for Economic and Social Affairs, 2023). This could explain why the few studies from India that have examined access to safe housing focus on large urban centers (Bhan & Jana, 2015; Haque et al., 2020; Nix et al., 2020), and why the government of India only reports on progress in urban communities (SDG India Index, 2023, 2024). Thus, filling gaps in knowledge regarding between and within district variations in housing quality, along with urban rural differences is critical as it can help policy makers understand which communities need to be prioritized.

Therefore, the purpose of this paper is to use the most recent all-India data to estimate the percentage of homes in each of India's districts that are a) completely made of finished materials, b) partially made of rudimentary materials, and c) entirely made of rudimentary materials. Next, we assessed how these district-level percentages changed between 2016 and 2021 to elucidate whether districts are improving or worsening in the provision of safe and adequate housing. We also examined changes in within-district between-community variations of housing quality between 2016 and 2021 and how these changes correspond with district-level changes in housing quality. We focus on this period to make our findings policy relevant, and conduct these analyses in both rural and urban communities.

## 2 Methods

### 2.1 Data and sampling strategy

We used data from the fourth and fifth round of India's National Family Health Survey (NFHS) from 2015 to 2016 and 2019 to 2021, respectively, to conduct this analysis. Hereafter, we refer to just the terminal year of each survey for simplicity. Both surveys are part of the Demographic and Health Surveys Program (DHS) and capture indicators relating to population health, nutrition, and well-being (Croft et al., 2023). As a part of this overall scope, the surveys include indicators pertaining to housing quality. Both surveys were designed such that communities – villages in rural areas and wards in urban communities – were selected first with probability proportional to size from districts within states. Then, households were randomly chosen from each community. A full description of the sampling strategy employed is available in the latest NFHS report (International Institute for Population Sciences, 2021).

### 2.2 Study population

The 2021 dataset contains information from 636,699 households nested 30,198 communities in 36 states and Union Territories (UTs). Information on housing structure material was collected from all households. However, we excluded those households that reported “other” when asked what material was used for a given structural component. For floors, 948 households reported “other”, and 2080 households reported “other” for walls. There were 6346 households that reported “other” for roof. We found that rural households were more likely to report “other” for all three structural components. Thus, the final number of households included from 2021 was 627,866, of which 158,391 were urban and 469,475 were rural.

The 2016 dataset contains information from 601,509 households nested in 28,522 communities in all 36 states and UTs. We again excluded those households that reported “other” when asked what material was used for a given structural component. For floors, 518 households reported “other”. For walls, 1964 reported “other”, and 19,591 reported “other” for roof. We found that rural households were more likely to report “other” for all three structural components. Thus, the number of households with complete information on each of the three structural components was 579,768, of which 171,652 were urban and 408,116 were rural.

In both 2016 and 2021, we found that the lower socioeconomic status households, as measured by household wealth and educational attainment of the household head, were more likely to report using “other materials” for the floor, walls, or roof. In both 2016 and 2021, rural households were far more likely to report using “other materials” for the floor, walls, or roof than urban households. However, we note that the overall number of households that reported using “other materials” for any of the structural components was a small share of the overall number of households in the sample. These results are presented in Supplementary Tables 1–3.

### 2.3 Outcomes

We examined three outcomes as a part of this study. These were finished housing, semi-finished housing, and rudimentary housing. Homes with finished walls, roof, and floors were considered finished. Homes with either raw or rudimentary floors, roof, or walls were defined as semi-finished. Finally, we define rudimentary housing as those homes that used raw or rudimentary materials for the floors, walls, and roof. The materials used for each classification are defined in Table 1.

**Table 1:** Description of materials used to define household floor, roof, and wall quality.

		**All-India**		**Urban**		**Rural**	
**Floor quality**		**2016**	**2021**	**2016**	**2021**	**2016**	**2021**

**Raw floor**	Mud/clay/earth | Sand | Dung	195964 (33.8, 33.6–33.9)	181886 (28.9, 28.8–29.0)	11422 (6.6, 6.5–6.8)	8585 (5.4, 5.3–5.5)	198347 (48.6, 48.4–48.8)	191057 (40.7, 40.5–40.8)
**Rudimentary floor**	Raw wood planks | Palm/bamboo | Brick | Stone	39273 (6.7, 6.7–6.8)	43412 (6.9, 6.8–6.9)	12804 (7.4, 7.3–7.5)	11713 (7.3, 7.2–7.5)	26121 (6.4, 6.3–6.5)	31336 (6.6, 6.6–6.7)
**Finished Floor**	Parquet/polished wood | Vinyl/asphalt | Ceramic tile | Cement | Carpet | Polished stone	344532 (59.4, 59.2–59.5)	402569 (64.1, 63.9–64.2)	147427 (85.9, 85.7–86.0)	138093 (87.1, 87.0–87.3)	183648 (45.0, 44.8–45.1)	247082 (52.6, 52.4–52.7)

**Roof quality**							

**No roof**		1173 (0.2, 0.1–0.2)	1357 (0.2, 0.2–0.2)	181 (0.1, 0.09–0.12)	137 (0.09, 0.07–0.1)	1041 (0.2, 0.2–0.3)	1318 (0.2, 0.2–0.3)
**Raw roof**	Thatch/palm/reed/grass | Mud | Sod/mud/grass | Plastic/polythene sheet	41549 (7.1, 7.1–7.2)	35266 (5.6, 5.5–5.7)	4065 (2.3, 2.2–2.4)	3246 (2.0, 1.9–2.1)	39924 (9.7, 9.6–9.8)	34709 (7.3, 7.3–7.5)
**Rudimentary roof**	Rustic mat | Palm/bamboo | Raw wood planks/timber | Unburnt brick | Loosely packed stone	25802 (4.4, 4.3–4.5)	39118 (6.2, 6.1–6.3)	4852 (2.8, 2.7–2.9)	6234 (3.9, 3.8–4.0)	21776 (5.3, 5.2–5.4)	34614 (7.3, 7.2–7.4)
**Finished roof**	Metal/GI | Wood | Calamine/cement fiber | Asbestos sheets | RCC/RBC/Cement/Concrete | Roofing shingles | Tiles | Slate | Burnt brick	511243 (88.1, 88.0–88.2)	552125 (87.9, 87.8–88.0)	162553 (94.7, 94.5–94.8)	148774 (93.9, 93.8–94.0)	345374 (84.6, 84.5–84.7)	398834 (84.9, 84.8–85.0)

**Wall quality**							

**No walls**		2024 (0.3, 0.3–0.4)	1673 (0.2 (0.2–0.3)	328 (0.2, 0.1–0.2)	174 (0.1, 0.09–0.1)	1777 (0.4, 0.4–0.5)	1616 (0.3, 0.3–0.4)
**Raw wall**	Cane/palm/trunks/bamboo | Mud | Grass/reeds/thatch	88923 (15.3, 15.2–15.4)	74639 (11.8, 11.8–11.9)	6353 (3.7, 3.6–3.8)	4297 (2.7, 2.6–2.8)	88488 (21.7, 21.6–21.8)	77260 (16.4, 16.3–16.5)
**Rudimentary wall**	Bamboo with mud | Stone with mud | Plywood | Cardboard | Unburnt brick | Raw wood/reused wood	42711 (7.3, 7.2–7.4)	47960 (7.6, 7.5–7.7)	5054 (2.9, 2.8–3.0)	6698 (4.2, 4.1–4.3)	39907 (9.7, 9.6–9.8)	43833 (9.3, 9.2–9.4)
**Finished wall**	Cement | Concrete | Stone with lime/cement | Burnt brick | Cement blocks | Wood/planks/shingles | GI/metal/Asbestos sheets	446109 (76.9, 76.8–77.0)	503595 (80.2, 80.1–80.3)	159918 (93.1, 93.0–93.2)	147222 (92.9, 92.8–93.1)	277944 (68.1, 67.9–68.2)	346766 (73.8, 73.7–73.9)

### 2.4 District geometry

A primary motivation of this study is to examine district-level variation in housing quality across India, and how this variation changed between NFHS-4 and NFHS-5. However, due to administrative reasons, India's sub-national geography is continuously updating with shifting district borders and geographies. There were 640 districts at the time of NFHS-4 and 707 districts at the time of NFHS-5. To compare changes in housing quality between these two survey rounds, we used an updated geometry of 720 districts. We used this updated 720 configuration instead of the 707 districts because the state of Andhra Pradesh (AP) created 13 new districts in April 2022 (Geographic Insights Lab, 2023). Including these new districts is important because none of the districts from AP included in NFHS-5 match with the updated district boundaries of that state, which would prohibit any meaningful interpretations regarding housing quality within AP.

We included the updated district geography from AP by first linking Assembly Constituency (AC) boundaries with the 707 district boundaries provided by the DHS Spatial Data Repository. (Spatial Data Repository) The AC linkage information was provided to us by the Chief Electoral Officer (CEO) of AP (Geographic Insights Lab, 2023). Representatives for each state legislature are elected from ACs, and AC boundaries are contained within the district boundaries for each state/UT. The AC shapefiles in AP can thus be used to create the updated district boundaries by using the linkage information provided by AP's CEO and dissolving the AC polygons to form the new district boundaries (Geographic Insights Lab, 2023). These steps allowed us to create 720 districts for NFHS-5. The final step was adjusting the 720-district shapefile to have the same external boundary of India as per the Survey of India's specifications. (Survey of India).

To link communities to the updated geometry, we did not make any changes to the PSU to district linkage for the 694 unchanged districts in NFHS-5 and 577 unchanged districts in NFHS-4 and used the PSU to district linkage present in the microdata. For the remaining 23 districts in AP for NFHS-5 and the 130 changed districts for NFHS-4, we used a PSU to district spatial join using the GPS coordinates for each surveyed PSU to assign them to the updated 720 district shapefile.

Finally, we note that there are 15 districts for which there was no rural data available in either 2016 or 2021, and 26 such urban districts in 2021. A list of these districts is provided in the Supplementary Table 4.

### 2.5 Statistical analysis

The NFHS data are structured such that households are nested in communities *j*, districts *k*, and states *l*. Given this nested structure, we used a Markov Chain Monte Carlo (MCMC) procedure to estimate a multilevel model to ascertain the prevalence of finished, semi-finished, and rudimentary housing given the novel district geometry used in this analysis. MCMC procedures follow a Bayesian approach in which prior knowledge is used to maximize a likelihood function (Browne, 2019). The estimated priors – in this case the default of iterated generalized least squares (which have been used in previously published work when examining geographic variation of outcomes in India (Jain, Ambade, et al., 2025; Jain, Kim, & Subramanian, 2025)) – were then used to apply MCMC to the model $logit\left({\mathrm{Pr}}_{ijkl}\right)=\beta_{0\;}+\left(u_{0jkl}+v_{0kl}+f_{0l}\right)$ for each of the three outcomes in each survey round where $\beta_{0\;}$ represents the constant, and $u_{0jkl}$, $v_{0kl}$, and $f_{0l}$ are the residual differentials for communities *j*, districts *k*, and states *l,* respectively. The residual differentials estimates were derived using the *runmlwin* command in Stata 18 (Leckie et al., 2012). The MCMC residuals were then used in equation (2) exp[$\beta_{0\;}+\left(u_{0jkl}+v_{0kl}+f_{0l}\right)]/[$ 1 + exp($\beta_{0\;}+\left(u_{0jkl}+v_{0kl}+f_{0l}\right)]$ to calculate the precision-weighted estimates for the share of each housing quality outcome in each community. Next, because communities are either entirely urban wards or rural villages, we found the average share of each housing quality indicator for rural and urban communities in each district. We took the rural and urban averages by district after doing the pooled analysis because the NFHS is designed to be district specific with representative numbers of rural and urban communities (We note that there are districts with both urban and rural communities. Thus a district could appear in both our rural and urban maps). Hence, we first derive the pooled estimates for each outcome to account for the possible influence/spillover between urban and rural communities in a district. The output from our MCMC models is presented in Supplementary Table 5. Finally, we show that the MCMC estimates are highly correlated with the crude estimates from the data in Supplementary Table 6, showing the goodness of fit of our models.

We used the district averages from 2016 to create decile cutoffs for each outcome. These cutoffs were used for the 2021 district averages to highlight how the share of each outcome changed over time for each district. We also calculated the difference between the 2016 and 2021 district-level averages for each outcome. We created seven categories of change based on these difference values. These were a decrease of more than 9.99 percentage points, a decrease between 4.99 and 9.99 percentage points, a decrease between 2.49 and 4.99 percentage points, a decrease of −2.49 percentage points to an increase of 2.49 percentage points (which we classify as no change), an increase between 2.49 and 4.99 percentage points, and increase between 4.99 and 9.99 percentage points, and an increase greater than 9.99 percentage points.

## 3 Results

### 3.1 Sample characteristics

The all-India share of finished housing was 55.9 % (95 % CI: 55.8–56.1) in 2016 and 60.1 % (95 % CI: 59.9–60.2) in 2021. Approximately 37.9 % (95 % CI: 37.7–38.0) of households were semi-finished in 2016, and 34.9 % (95 % CI: 34.8–35.1) of households were semi-finished in 2021. The share of rudimentary housing was approximately 6.1 % (95 % CI: 6.0–6.2) in 2016 and 4.9 % (95 % CI: 4.9–5.0) in 2021.

The rural share of finished housing was 41.3 % (95 % CI: 41.1–41.4) in 2016 and 48.5 % (95 % CI: 48.3–48.6) in 2021. The rural share of semi-finished homes was 49.8 % (95 % CI: 49.7–50.0) in 2016 and 44.5 % (95 % CI: 44.4–44.7) in 2021. The rural share of rudimentary housing was 8.8 % (95 % CI: 8.7–8.9) in 2016 and was 6.9 % (95 % CI: 6.8–6.9) in 2021.

The urban share of finished housing was 82.9 % (95 % CI: 82.7–83.1) in 2016 and 83.2 % (95 % CI: 83.1–83.4) in 2021. The share of semi-finished housing in urban areas was 15.9 % (95 % CI: 15.8–16.1) in 2016 and 15.6 % (95 % CI: 15.5–15.8) in 2021. The urban share of rudimentary housing was 1.1 % (95 % CI: 1.0–1.1) in 2016 and 1.1 % (95 % CI: 1.0–1.1) in 2021.

In both 2016 and 2021, we found that the poorest households and those with the least education were the most likely to live in either rudimentary or semi-finished housing, and the least likely to live in finished housing. Those belonging to Scheduled Tribes were the most likely to live in rudimentary or semi-finished housing, and the least likely to live in finished housing. We did not find any consistent patterns of housing quality across religious groups. These results are presented in Supplementary Table 7.

### 3.2 Geographic variation in housing quality

**Fig. 1:**
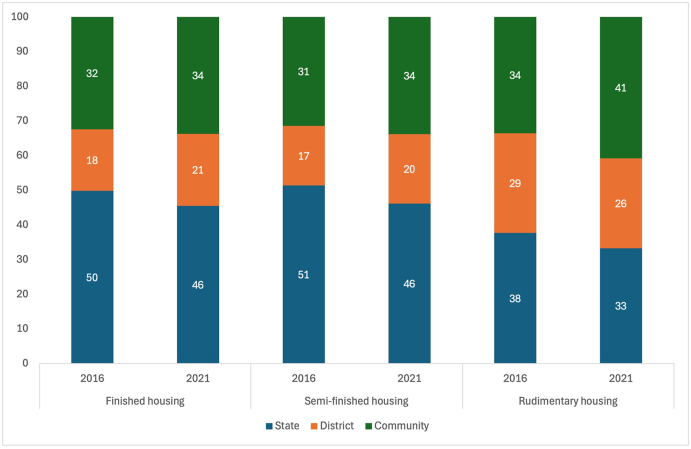
Geographic variance partitioned between states, districts, and communities for finished housing, semi-finished housing, an rudimentary housing in 2016 and 2021. Each residual is assumed to be normally distributed with a mean of zero and a variance of $u_{0jkl}$ ∼ N(0, $\sigma_{u0}^{2}$), $v_{0kl}$ ∼ N(0, $\sigma_{v0}^{2}$), and $f_{0l}$ ∼ N(0, $\sigma_{f0}^{2}$), allowing us to calculate the proportion of variation in each outcome attributable to clusters, districts, and states by dividing the variance of a given level by the total geographic variation (i.e., for the cluster level, $\sigma_{u0}^{2}$/($\sigma_{u0}^{2}$ + $\sigma_{v0}^{2}$ + $\sigma_{f0}^{2}$) X 100).

We used the model residuals to assess the variation in each outcome between states, districts, and communities. Overall, we found considerable variation within districts and between communities – both urban and rural – as demonstrated by the partitioned geographic variation, which is shown in Fig. 1 and Supplementary Table 5. For each of the three outcomes, the variation between communities was higher than the variation between districts in both 2016 and 2021. The variation between states was higher than the variation between communities in both 2016 and 2021 for all three outcomes. The only exception to this was for rudimentary housing in 2021 when the variation was greater between communities (41 %) than states (33 %).

### 3.3 Between-district variation in rural and urban housing quality

**Fig. 2:**
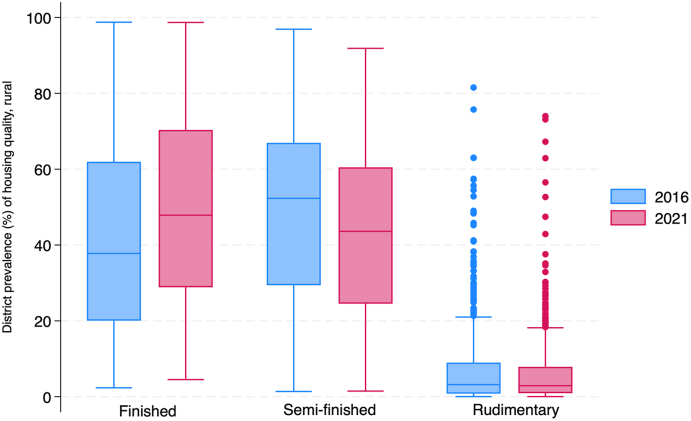
Rural distribution of finished*,* semi-finished*,* and rudimentary housing in 2016 (blue) and 2021 (red). The upper and lower whiskers represent minimum and maximum values, respectively. The upper outline of the box depicts the 75th percentile and the lower outline the 25th percentile. The solid line within the box shows the median (50th percentile).

**Fig. 3:**
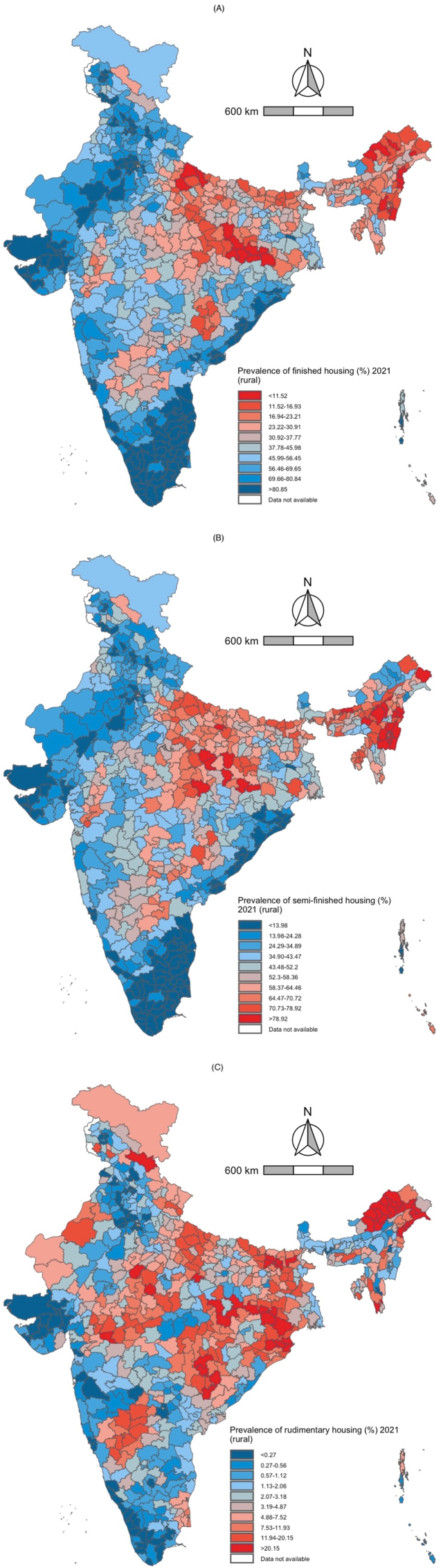
Rural India maps depicting the district-level prevalence of three indicators of housing quality in 2021. A. Finished housing B. Semi-finished housing C. Rudimentary housing. Decile cutoff values are based on the prevalence of each outcome in 2016 to highlight changes in district prevalence between the two time periods.

**Fig. 4:**
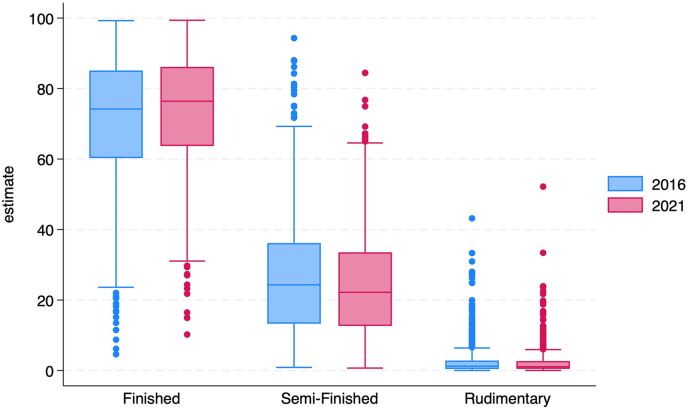
Urban distribution of finished*,* semi-finished*,* and rudimentary housing in 2016 (blue) and 2021 (red). The upper and lower whiskers represent minimum and maximum values, respectively. The upper outline of the box depicts the 75th percentile and the lower outline the 25th percentile. The solid line within the box shows the median (50th percentile).

**Fig. 5:**
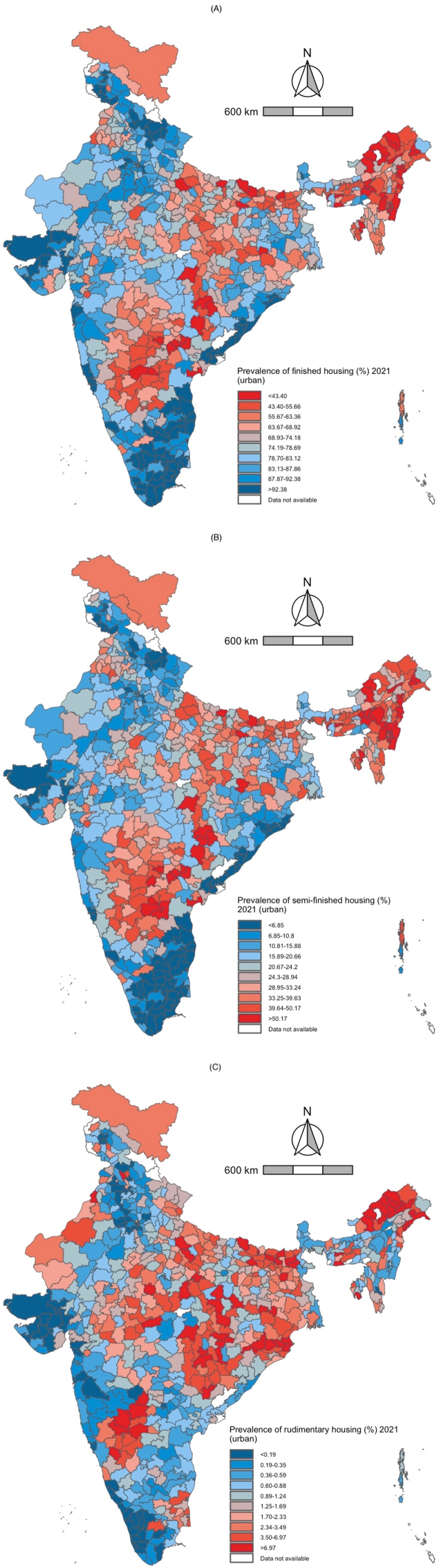
Urban India maps depicting the district-level prevalence of three indicators of housing quality in 2021. A. Finished housing B. Semi-finished housing C. Rudimentary housing. Decile cutoff values are based on the prevalence of each outcome in 2016 to highlight changes in district prevalence between the two time periods.

In 2016, the median district prevalence of finished housing in rural India was 37.7 %, and 47.9 % in 2021. The interquartile range (IQR) for finished housing was approximately 41 in both years. In urban India, the median district prevalence of finished housing was 74.2 %, and 76.4 % in 2021. The interquartile range (IQR) for finished housing in urban India decreased slightly between 2016 and 2021. These results are presented in Fig. 2, Fig. 3, Fig. 4, Fig. 5 and Supplementary Figs. 1 and 2.

For semi-finished housing, the median district prevalence in rural India was 52.3 % in 2016 and 43.6 % in 2021. The interquartile range (IQR) for semi-finished housing decreased slightly between 2016 and 2021. In urban India, the median district prevalence of semi-finished housing was 24.3 % in 2016 and 22.2 % in 2021. The interquartile range (IQR) for semi-finished housing in urban India decreased slightly between 2016 and 2021. These results are presented in Fig. 2, Fig. 3, Fig. 4, Fig. 5 and Supplementary Figs. 1 and 2.

In 2016, the median district prevalence of rudimentary housing in rural India was 3.2 % and 2.9 % in 2021. The interquartile range (IQR) for rudimentary housing decreased slightly between 2016 and 2021. In urban India, the median district prevalence of rudimentary housing was 1.2 % and 1.1 % in 2021. The interquartile range (IQR) for rudimentary housing in urban India decreased slightly between 2016 and 2021.These results are presented in Fig. 2, Fig. 3, Fig. 4, Fig. 5 and Supplementary Figs. 1 and 2.

### 3.4 Rural and urban changes in district-level prevalence between 2016 and 2021

**Fig. 6:**
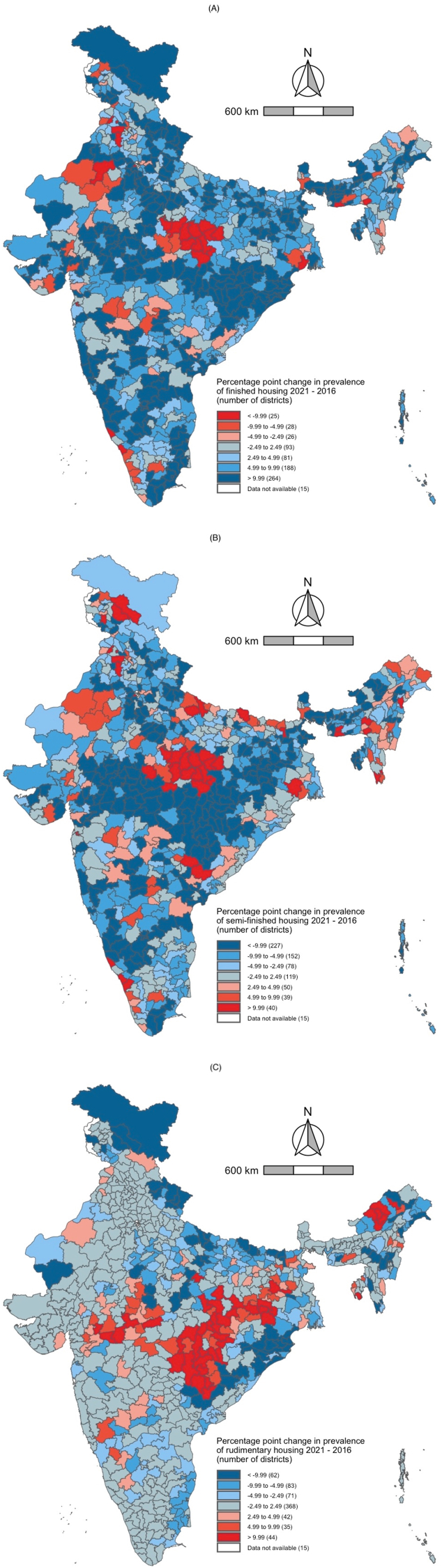
Maps for rural India depicting the number of districts that experienced increases or decreases in prevalence of (A) finished housing, (B) semi-finished housing, and (C) rudimentary housing between 2016 and 2021.

**Fig. 7:**
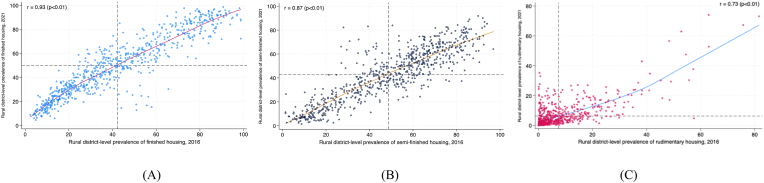
Scatter plot describing the correlation between the rural district-level prevalence of three indicators of housing quality in 2016 and 2021. The r-value is the Pearson correlation coefficient (p-value). A. Finished housing B. Semi-finished housing C. Rudimentary housing.

In rural India, 93 districts experienced no change in the share of finished housing between 2016 and 2021. We found that 264 districts experienced an increase of more than 9.99 percentage points in the share of finished housing between 2016 and 2021, while 25 districts experienced a decrease of more than 9.99 percentage points in the share of finished housing between 2016 and 2021. We found that there were 119 districts that experienced no change in the share of semi-finished housing between 2016 and 2021. However, 40 districts experienced an increase of more than 9.99 percentage points in the share of semi-finished housing between 2016 and 2021, while 227 districts experienced a decrease of more than 9.99 percentage points in the share of semi-finished housing between 2016 and 2021. There were 368 districts that experienced no change in the share of rudimentary housing between 2016 and 2021. However, the share of rudimentary housing increased by more than 9.99 percentage points in 44 districts. The share of rudimentary housing decreased by more than 9.99 percentage points in 62 districts. Overall, we found that the districts with a high prevalence of each outcome in 2016 still had a high prevalence in 2021. These results are presented in Fig. 6, Fig. 7.

**Fig. 8:**
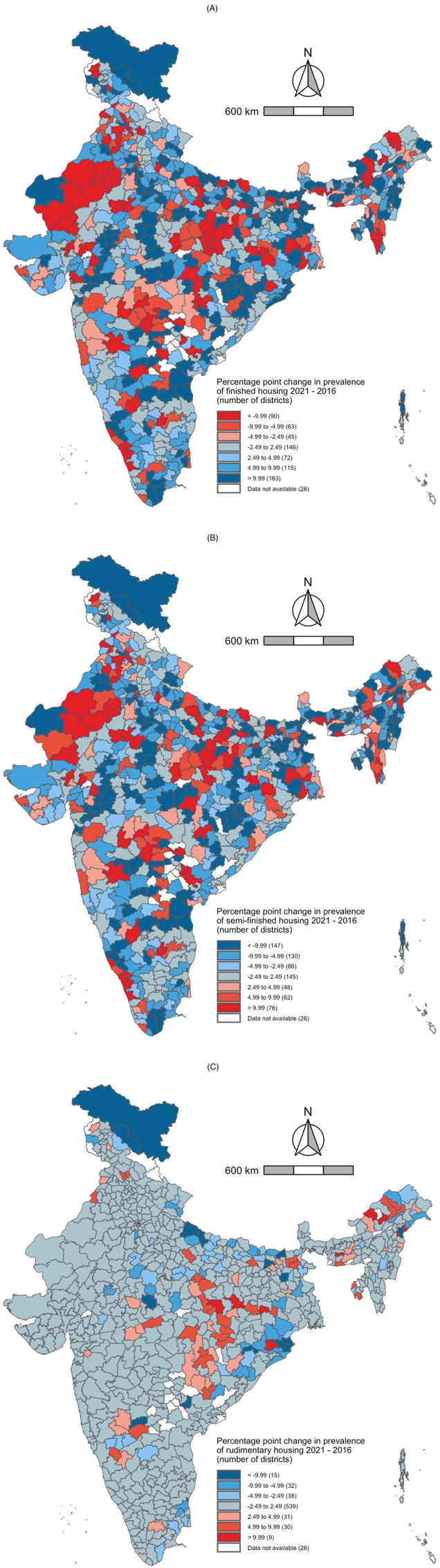
Maps for urban India depicting the number of districts that experienced increases or decreases in prevalence of (A) finished housing, (B) semi-finished housing, and (C) rudimentary housing between 2016 and 2021.

**Fig. 9:**
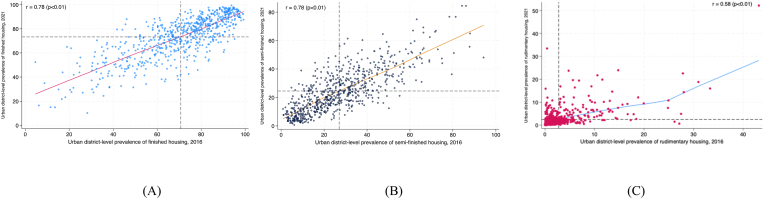
Scatter plot describing the correlation between the urban district-level prevalence of three indicators of housing quality in 2016 and 2021. The r-value is the Pearson correlation coefficient (p-value). A. Finished housing B. Semi-finished housing C. Rudimentary housing.

In urban India, 146 districts experienced no change in the share of finished housing between 2016 and 2021. The share of finished housing increased by more than 9.99 percentage points between 2016 and 2021 in 163 districts. The share of finished housing decreased by more than 9.99 percentage points between 2016 and 2021 in 90 districts. We found no change in the share of semi-finished housing in 145 districts between 2016 and 2021. The share of semi-finished housing increased by more than 9.99 percentage points in 76 districts between 2016 and 2021 but decreased by more than 9.99 percentage points in 147 districts during the same period. Finally, there was no change in the share of rudimentary housing in 539 districts between 2016 and 2021. The share of rudimentary housing increased by more than 9.99 percentage points in nine districts between 2016 and 2021. The share of rudimentary housing decreased by more than 9.99 percentage points in 15 districts between 2016 and 2021. Overall, we found that the districts with a high prevalence of each outcome in 2016 still had a high prevalence in 2021. These results are presented in Fig. 8, Fig. 9.

## 4 Discussion

Our study adds four novel and important insights into the state of housing quality in rural and urban India. First, housing quality, as measured by finished, semi-finished, and rudimentary housing, improved slightly in rural communities between 2016 and 2021. There were very small improvements in housing quality in urban communities between 2016 and 2021. Second, we found a larger share of variation in each outcome between communities than between districts in both 2016 and 2021. Third, housing quality varied considerably between districts for both rural and urban communities. Fourth, district-level housing quality improved throughout many rural and urban communities. However, it also worsened in many cases, underscoring persistent between-district inequality in housing quality outcomes throughout India. And the districts that were the worst off in 2016 in terms of rural and urban housing quality remained the worst off in 2021 for all three outcomes.

There are four data limitations necessary to consider when interpreting these results. First, as noted in the methods, 54 communities from 2016 were not included because we were unable to link them with a current district. Thus, our results are not representative of those communities. Second, changes to the district geometry of Andhra Pradesh means that we included 26 districts from this state even though the data were only collected from 13, which could mean that the results from these districts are underpowered. However, a previously published study that employed this new district geometry from Andhra Pradesh shows that the standard errors of estimates from these districts are small, an indication that the estimates are precise (Jain et al., 2024). Third, the Covid-19 pandemic disrupted India's construction sector in myriad ways (Rani et al., 2022). However, the NFHS did not have sufficient data for us to explore this relationship as a part of this study. Fourth, the NFHS does not survey the same communities between survey waves and thus does not contain data on which communities transitioned from being rural to urban between 2016 and 2021. Therefore, we were unable to assess how this might influence our outcomes.

Despite these limitations, our findings are policy relevant for several reasons. First, we found that a large share of geographic variation in the three measures of housing quality is between communities within districts. This is an important finding because currently, the SDGs only track progress towards safe housing in urban communities (United Nations Department for Economic and Social Affairs, 2023). Yet, as of 2021, less than half of the homes in rural communities were constructed entirely of finished materials. And in some districts, the share of finished housing was much lower. We also show that rudimentary housing is much more common in rural communities than urban ones. These findings underscore the importance of tracking housing quality progress within districts and between communities. Doing so highlights the fact that a district's rural communities are lagging urban communities in terms of housing quality.

Programs such as the PMAY have achieved modest success in improving housing quality in rural communities throughout India (Sharma, 2019). However, our results show that there is still much room for improvement. Improper identification of beneficiaries, delayed fund disbursement, and the inadequate provision of building materials are some factors that could undermine programs such as PMAY from improving housing quality in rural areas (Alam et al., 2022). Furthermore, in other parts of India, subsidized housing lotteries have helped people build better quality homes (Kumar, 2021). A redevelopment project in a slum in Ahmedabad also showed improvements in housing quality (Vaid, 2021). There are other ways that PMAY could be further improved to help more people gain access to finished housing. For example, there is very little difference in funding available from PMAY for household construction in plain versus mountainous areas (Housing for All Mission, 2021). Increasing the amount provided to rural households in mountainous communities could increase the share of finished homes in these areas. Additionally, states have different ways of verifying whether an individual owns the plot of land they live on, a key eligibility criterion under PMAY. The PMAY systems do not account for these different methods of verification. Thus, people could be wrongly deemed ineligible for PMAY benefits thus preventing them from constructing a finished home. Creating uniform methods of land ownership verification could help improve finished housing coverage in India.

Second, the share of urban households made entirely of finished materials was approximately 83 % in 2016 and 2021. This highlights the fact that housing quality is much better in urban communities throughout India. The very small increase (less than one percentage point) between 2016 and 2021 was possibly due to a ceiling effect. It is also possible that the Covid-19 pandemic, which resulted in labor shortages, disruptions of building material supply chains, and decreased construction productivity also contributed to very small gains in the share of finished housing in urban India (Rani et al., 2022). We also found that the share of finished housing was much lower than the national average in certain districts. In three districts of Assam for example, West Karbi Anglong, Nagaon, and Charaideo, the share of finished housing was less than 50 % in 2021. This was also the case in Sitapur and Hardoi, two districts in Uttar Pradesh. High construction costs, land use restrictions, a dearth of skilled labor, overly bureaucratic approval processes, and a reliance on outdated construction technologies are some of the factors that have contributed to slow improvements in housing quality in urban India (Strategy for New India, 2018). Policies should be designed with these barriers in mind. Policies already in place also need to be better enforced. For example, cities such as New Delhi should enforce housing quality guidelines that have been enacted to ensure residents live in good quality homes (Ahmad et al., 2025). Similarly, cities across India are redeveloping slums. However, low-quality materials have been reportedly used in these projects (Vaid, 2021). Thus, mechanisms of oversight, monitoring, and building code enforcement are needed to ensure that efforts aimed at improving housing quality are actually working (Vaid, 2021). In urban areas, granting legal tenure security can also motivate residents to invest in building better quality homes (Haque et al., 2020a; Nakamura, 2017), a policy lever that could be used to improve housing quality in urban India. The combination of these policy efforts is important given that 40 % of India's population is expected to live in urban areas by 2040 (Kouamé, 2024).

Third, our findings underscore the need for strengthened institutional capacity to consistently monitor and track safe housing access over time within India. Programs such as PMAY, Swachh Bharat Abhiyan, and the Pradhan Mantri Ujjwala Yojana have helped improve the quality of millions of homes in India through the provision of safe building materials, toilets, and clean cooking fuel, respectively. These programs notwithstanding, forces such as climate change can quickly undo progress, something the government needs to track over time. Extreme flooding affected Madhya Pradesh, Jammu & Kashmir, West Bengal, Chhattisgarh, Kerala, and Bihar between 2016 and 2021 (Kerala flood, 2019; Dash; Flood-like situation in 4 Chhattisgarh, 2020; Many flee North Dinajpur as, 2019). Our results show districts within each of these states either experienced increases in rudimentary housing or decreases in finished housing between this time period. Furthermore, our results show that as of 2021, almost 35 % of homes in India are still only semi-finished. Prior studies have shown that those living in semi-finished homes or homes made entirely of rudimentary materials are vulnerable to the effects of climate change in parts of India (Akhter Feroz, 2012; Ghosh & Ghosal, 2020; Sam et al., 2017). Households made with more permanent materials have been found to better withstand the impacts of flooding in India (Rumbach & Shirgaokar, 2017). Studies conducted in other contexts outside India highlight the importance of recovery and resilience planning as a way to protect those living in low quality homes (Li et al., 2023). Communities in the Himalayan foothills and coastal cities such as Kolkata are particularly vulnerable to flooding (Balica et al., 2012; Roy et al., 2021), something that will likely get worse with climate change. Policy makers should rely on novel tools being developed to better understand housing resiliency to flooding (Sen et al., 2025). These tools can then help local governments to build systems that track changes in housing quality over time while implementing climate resilient housing solutions and policies.

Ensuring safe and adequate housing throughout India is imperative from a population health perspective. Building homes with finished floors can help reduce the burden of enteric and parasitic infections (Legge et al., 2023). And in India, indoor temperature is more stable and comfortable in finished homes (Nix et al., 2015). Extreme heat and cold are both associated with an increased risk of mortality in India (McMichael et al., 2008; Nori-Sarma et al., 2019). Building safe and adequate homes can help protect people from these extreme temperatures. Furthermore, homes that are not adequately heated are more likely to have mold, a consequence of dampness (Heseltine and Rosen, 2009; Oreszczyn et al., 2006), which is associated with an increased risk of several respiratory outcomes, including asthma, wheeze, cough, respiratory infections and upper respiratory symptoms (IoM, 2004; Mendell et al., 2011). It should be noted, however, that identifying the specific attributes of housing that correspond to health risk requires assessments of housing attributes and occupant behavior that are more complicated than the degree of ‘finishing’ (World Health Organization, 2018). Thus, both unfinished and finished homes can vary significantly in their healthy attributes.

In conclusion, our paper shows that while the all-India share of finished housing has increased over the past few decades, progress has been far more inconsistent between and within districts. This is true for both rural and urban communities. Housing policies need to be reflective the challenges unique to rural and urban communities that are barriers to safe housing access while also being climate resilient and adaptive. Finally, our study underscores the importance of including rural targets towards safe housing.

## CRediT authorship contribution statement

**Anoop Jain:** Writing – original draft, Formal analysis, Data curation, Conceptualization. **Gary Adamkiewicz:** Writing – review & editing, Investigation, Conceptualization. **Rockli Kim:** Writing – review & editing, Supervision, Investigation, Conceptualization. **S.V. Subramanian:** Writing – review & editing, Supervision, Investigation, Conceptualization.

## Ethics approval and consent to participate

Not applicable.

## Consent for publication

Not applicable.

## Availability of data and materials

The datasets used and/or analyzed during the current study are available from the corresponding author upon reasonable request.

## Funding

This work was supported by the Bill & Melinda Gates Foundation, INV-002992. The funder had no role in the design and conduct of the study; collection, management, analysis, and interpretation of the data; preparation, review, or approval of the manuscript; and decision to submit the manuscript for publication.

## Competing interests

The authors declare that they have no competing interests.

## Data Availability

The authors do not have permission to share data.
