# Supplementary material for: Prevalence of housing structure and quality indicators in India: An assessment of changes across 720 districts between 2016 and 2021
Source: SSM Popul Health. 2025 Dec 14;33:101899. doi: 10.1016/j.ssmph.2025.101899 (PMC12857276; doi:10.1016/j.ssmph.2025.101899)
Supplement: Multimedia component 1 [file mmc1.docx]

**Supplementary online content**

| **Table of Contents** | | |
| --- | --- | --- |
| **Table/Figure** | **Title** | **Page** |
| **eTable1** | Comparison of households that reported using “other materials” for either wall, floor, or roof versus those that reported using either natural, rudimentary, or finished materials in 2016 (NFHS-4) and 2021 (NFHS-5). Comparisons are by whether the household was in an urban or rural community. Values presented are n (number of households) and percent (%) of household location that reported using either natural, rudimentary, and finished materials or “other materials” | 2 |
| **eTable2** | Comparison of households that reported using “other materials” for either wall, floor, or roof versus those that reported using either natural, rudimentary, or finished materials in 2021 (NFHS-5). Comparisons are by household wealth quintile and educational attainment of the head of household. Values presented are n (number of households) and percent (%) of each socioeconomic strata that reported using either natural, rudimentary, and finished materials or “other materials” | 3 |
| **eTable3** | Comparison of households that reported using “other materials” for either wall, floor, or roof versus those that reported using either natural, rudimentary, or finished materials in 2016 (NFHS-4) and 2021 (NFHS-5). Comparisons are by whether the household was in an urban or rural community. Values presented are n (number of households) and percent (%) of household location that reported using either natural, rudimentary, and finished materials or “other materials” | 4 |
| **eTable4** | List of districts with no housing quality data by year and by urban/rural | 5 |
| **eTable5** | Markov Chain Monte Carlo (MCMC) model output for rudimentary housing, finished housing, and semi-finished housing in 2016 and 2021 | 6 |
| **eTable6** | Pearson correlation value (p-value) between Markov Chain Monte Carlo estimates and crude estimates for the district-level prevalence of three indicators of housing quality – finished housing, semi-finished housing, and rudimentary housing | 7 |
| **eTable 7** | Percentage of households by wealth quintile, caste, education (of household head), and caste living in rudimentary, semi-finished, and finished housing in 2016 and 2021 | 8 |
| **efigure 1** | Rural India maps depicting the district-level prevalence of three indicators of housing quality in 2016. A. Finished housing B. Semi-finished housing C. Rudimentary housing. Decile cutoff values are based on the prevalence of each outcome in 2016. | 9 |
| **efigure 2** | Urban India maps depicting the district-level prevalence of three indicators of housing quality in 2016. A. Finished housing B. Semi-finished housing C. Rudimentary housing. Decile cutoff values are based on the prevalence of each outcome in 2016. | 10 |

**Supplementary Table 1:** Comparison of households that reported using “other materials” for either wall, floor, or roof versus those that reported using either natural, rudimentary, or finished materials in 2016 (NFHS-4). Comparisons are by household wealth quintile and educational attainment of the head of household. Values presented are n (number of households) and percent (%) of each socioeconomic strata that reported using either natural, rudimentary, and finished materials or “other materials”

| **2016** | | | | | |  |
| --- | --- | --- | --- | --- | --- | --- |
| **Walls** | **Lowest** | **Low** | **Middle** | **High** | **Highest** | |
| **No walls/natural/rudimentary/finished materials** | 131236 (21.89%) | 129603 (21.62%) | 121436 (20.25%) | 112008 (18.68%) | 105262 (17.56%) | |
| **Other materials** | 492 (25.1%) | 353 (18%) | 481 (24.5%) | 410 (20.9%) | 228 (11.6%) | |
| **Floors** |  |  |  |  |  | |
| **Natural/rudimentary/finished materials** | 131604 (21.9%) | 129873 (21.61%) | 121799 (20.27%) | 112334 (18.69%) | 105381 (17.53%) | |
| **Other materials** | 124 (23.9%) | 83 (16%) | 118 (22.8%) | 84 (16.2%) | 109 (21%) | |
| **Roof** |  |  |  |  |  | |
| **No roof/natural/rudimentary/finished materials** | 122792 (21.1%) | 125116 (21.5%) | 118996 (20.45%) | 110435 (18.98%) | 104579 (17.97%) | |
| **Other materials** | 8936 (45.6%) | 4840 (24.7%) | 2921 (14.9%) | 1983 (10.1%) | 911 (4.7%) | |
| **Walls** | **No education** | **Up to fifth grade** | **Up to eighth grade** | **Up to twelfth grade** | **Above twelfth grade** | |
| **No walls/natural/rudimentary/finished materials** | 187883 (31.37%) | 109345 (18.26%) | 91956 (15.36%) | 152159 (25.41%) | 57519 (9.6%) | |
| **Other materials** | 546 (27.8%) | 499 (25.5%) | 361 (18.4%) | 430 (21.9%) | 125 (6.4%) | |
| **Floors** |  |  |  |  |  | |
| **Natural/rudimentary/finished materials** | 188284 (31.36%) | 109740 (18.28%) | 92249 (15.37%) | 152454 (25.4%) | 57579 (9.59%) | |
| **Other materials** | 145 (28.1%) | 104 (20.1%) | 68 (13.2%) | 135 (26.1%) | 65 (12.6%) | |
| **Roof** |  |  |  |  |  | |
| **No roof/natural/rudimentary/finished materials** | 180858 (31.11%) | 105921 (18.22%) | 89269 (15.36%) | 148532 (25.55%) | 56690 (9.75%) | |
| **Other materials** | 7571 (38.7%) | 3923 (20.1%) | 3048 (15.6%) | 4057 (20.8%) | 954 (4.9%) | |

**Supplementary Table 2:** Comparison of households that reported using “other materials” for either wall, floor, or roof versus those that reported using either natural, rudimentary, or finished materials in 2021 (NFHS-5). Comparisons are by household wealth quintile and educational attainment of the head of household. Values presented are n (number of households) and percent (%) of each socioeconomic strata that reported using either natural, rudimentary, and finished materials or “other materials”

| **2021** | | | | | | |
| --- | --- | --- | --- | --- | --- | --- |
| **Walls** | **Lowest** | **Low** | **Middle** | **High** | **Highest** |  |
| **No walls/natural/rudimentary/finished materials** | 148148 (23.34%) | 140589 (22.15%) | 128589 (20.26%) | 114707 (18.07%) | 102586 (16.16%) |  |
| **Other materials** | 624 (30%) | 563 (27.1%) | 468 (22.5%) | 295 (14.2%) | 130 (6.3%) |  |
| **Floors** |  |  |  |  |  |  |
| **Natural/rudimentary/finished materials** | 148516 (23.36%) | 140863 (22.16%) | 128869 (20.27%) | 114874 (18.07%) | 102629 (16.14%) |  |
| **Other materials** | 256 (27%) | 289 (30.5%) | 188 (19.8%) | 128 (13.5%) | 87 (9.2%) |  |
| **Roof** |  |  |  |  |  |  |
| **No roof/natural/rudimentary/finished materials** | 146124 (23.18%) | 139392 (22.11%) | 127946 (20.3%) | 114391 (18.15%) | 102500 (16.26%) |  |
| **Other materials** | 2648 (41.7%) | 1760 (27.7%) | 1111 (17.5%) | 611 (9.6%) | 216 (3.4%) |  |
| **Walls** | **No education** | **Up to fifth grade** | **Up to eighth grade** | **Up to twelfth grade** | **Above twelfth grade** |  |
| **No walls/natural/rudimentary/finished materials** | 191029 (30.13%) | 114838 (18.11%) | 99295 (15.66%) | 168923 (26.64%) | 59987 (9.46%) |  |
| **Other materials** | 568 (27.4%) | 488 (23.6%) | 371 (17.9%) | 520 (25.1%) | 123 (5.9%) |  |
| **Floors** |  |  |  |  |  |  |
| **Natural/rudimentary/finished materials** | 191318 (30.12%) | 115131 (18.13%) | 99492 (15.66%) | 169227 (26.64%) | 60026 (9.45%) |  |
| **Other materials** | 279 (29.4%) | 195 (20.6%) | 174 (18.4%) | 216 (22.8%) | 84 (8.9%) |  |
| **Roof** |  |  |  |  |  |  |
| **No roof/natural/rudimentary/finished materials** | 189305 (30.06%) | 113895 (18.08%) | 98650 (15.66%) | 168121 (26.69%) | 59836 (9.5%) |  |
| **Other materials** | 2292 (36.2%) | 1431 (22.6%) | 1016 (16%) | 1322 (20.9%) | 274 (4.3%) |  |

**Supplementary Table 3:** Comparison of households that reported using “other materials” for either wall, floor, or roof versus those that reported using either natural, rudimentary, or finished materials in 2016 (NFHS-4) and 2021 (NFHS-5). Comparisons are by whether the household was in an urban or rural community. Values presented are n (number of households) and percent (%) of household location that reported using either natural, rudimentary, and finished materials or “other materials”

|  | **2016** | |
| --- | --- | --- |
| **Walls** | **Urban** | **Rural** |
| **No walls/natural/rudimentary/finished materials** | 175218 (29.23%) | 424327 (70.77%) |
| **Other materials** | 728 (37.1%) | 1236 (62.9%) |
| **Floors** |  |  |
| **Natural/rudimentary/finished materials** | 175759 (29.24%) | 425232 (70.76%) |
| **Other materials** | 187 (36.1%) | 331 (63.9%) |
| **Roof** |  |  |
| **No roof/natural/rudimentary/finished materials** | 172496 (29.64%) | 409422 (70.36%) |
| **Other materials** | 3450 (17.6%) | 16141 (82.4%) |
| **Walls** | **2021** | |
| **No walls/natural/rudimentary/finished materials** | 159580 (25.15%) | 475039 (74.85%) |
| **Other materials** | 558 (26.8%) | 1522 (73.2%) |
| **Floors** |  |  |
| **Natural/rudimentary/finished materials** | 159904 (25.15%) | 475847 (74.85%) |
| **Other materials** | 234 (24.7%) | 714 (75.3%) |
| **Roof** |  |  |
| **No roof/natural/rudimentary/finished materials** | 159093 (25.24%) | 471260 (74.76%) |
| **Other materials** | 1045 (16.5%) | 5301 (83.5%) |

**Supplementary Table 4:** List of districts with no housing quality data by year and by urban/rural

| **Missing data for both 2016 and 2021** | | **Missing data for 2016** | | **Missing data for 2021** | |  |
| --- | --- | --- | --- | --- | --- | --- |
| **Rural** | | **Rural** | | **Urban** | |  |
| **District** | **State** | **District** | **State** | **District** | **State** | |
| Kolkata | West Bengal | East | Nct Of Delhi | Alluri Sitharama Raju | Andhra Pradesh | |
| Mumbai Suburban | Maharashtra | North | Nct Of Delhi | Dr. B.R. Ambedkar Konaseema | Andhra Pradesh | |
| Mumbai | Maharashtra | North East | Nct Of Delhi | Jabalpur | Madhya Pradesh | |
| Chennai | Tamil Nadu | South | Nct Of Delhi |  |  | |
| Yanam | Puducherry | West | Nct Of Delhi |  |  | |
| New Delhi | Nct Of Delhi | **Urban** | |  |  | |
| Shahdara | Nct Of Delhi | Biswanath | Assam |  |  | |
| South East | Nct Of Delhi | South Salmara Mancachar | Assam |  |  | |
| Hyderabad | Telangana | Gariyaband | Chhattisgarh |  |  | |
| Mahe | Puducherry | Mahisagar | Gujarat |  |  | |
| **Urban** | | Jagitial | Telangana |  |  | |
| Lahul & Spiti | Himachal Pradesh | Jangoan | Telangana |  |  | |
| Kinnaur | Himachal Pradesh | Jayashankar Bhupalapally | Telangana |  |  | |
| Kra Daadi | Arunachal Pradesh | Jogulamba Gadwal | Telangana |  |  | |
| Majuli | Assam | Komaram Bheem Asifabad | Telangana |  |  | |
| East Jantia Hills | Meghalaya | Nagarkurnool | Telangana |  |  | |
| South West Garo Hills | Meghalaya | Siddipet | Telangana |  |  | |
| South West Khasi Hills | Meghalaya | Suryapet | Telangana |  |  | |
| Nicobars | Andaman & Nicobar Islands | Vikarabad | Telangana |  |  | |
|  |  | Yadadri Bhuvanagiri | Telangana |  |  | |
|  |  | Khowai | Tripura |  |  | |

**Supplementary table 5:** Markov Chain Monte Carlo (MCMC) model output for rudimentary housing, finished housing, and semi-finished housing in 2016 and 2021

| **Survey/outcome** | **Geographic level** | **Coefficient** | **Std Dev** | **Effective Sample Size** | **Credible interval** | **Other MCMC** | |
| --- | --- | --- | --- | --- | --- | --- | --- |
| **NFHS 4 finished housing** | **Constant** | 0.42 | 0.28 | 6631 | (-0.13, 0.97) | dbar | 523848.7 |
|  | **State** | 2.75 | 0.77 | 899 | (1.59, 4.59) | thetabar | 501425.9 |
|  | **District** | 0.98 | 0.06 | 1911 | (0.87, 1.10) | effective no. of pars | 22422.7 |
|  | **Cluster** | 1.79 | 0.02 | 1424 | (1.75, 1.84) | B DIC | 546271.4 |
| **NFHS 5 finished housing** | **Constant** | 0.61 | 0.23 | 6484 | (0.16, 1.07) | dbar | 598931.6 |
|  | **State** | 1.82 | 0.49 | 5159 | (1.09, 2.97) | thetabar | 575590 |
|  | **District** | 0.83 | 0.05 | 1533 | (0.74, 0.93) | effective no. of pars | 23341.6 |
|  | **Cluster** | 1.35 | 0.02 | 1352 | (1.32, 1.39) | B DIC | 622273.2 |
| **NFHS 4 semi-finished housing** | **Constant** | -0.83 | 0.25 | 6423 | (-1.32, -0.35) | dbar | 567139.2 |
|  | **State** | 2.09 | 0.58 | 2148 | (1.23, 3.49) | thetabar | 545296.6 |
|  | **District** | 0.7 | 0.04 | 3000 | (0.62, 0.79) | effective no. of pars | 21842.5 |
|  | **Cluster** | 1.28 | 0.02 | 1360 | (1.25, 1.31) | B DIC | 588981.7 |
| **NFHS 5 semi-finished housing** | **Constant** | -0.94 | 0.2 | 6833 | (-1.35, -0.54) | dbar | 627776.2 |
|  | **State** | 1.43 | 0.39 | 862 | (0.84, 2.38) | thetabar | 605180.5 |
|  | **District** | 0.62 | 0.04 | 2531 | (0.55, 0.69) | effective no. of pars | 22595.6 |
|  | **Cluster** | 1.05 | 0.01 | 1182 | (1.03, 1.08) | B DIC | 650371.8 |
| **NFHS 4 rudimentary housing** | **Constant** | -4.51 | 0.26 | 2344 | (-5.04, -4.02) | dbar | 196578.1 |
|  | **State** | 2.03 | 0.64 | 937 | (1.11, 3.61) | thetabar | 185035.3 |
|  | **District** | 1.55 | 0.1 | 2012 | (1.36, 1.77) | effective no. of pars | 11542.8 |
|  | **Cluster** | 1.81 | 0.04 | 343 | (1.74, 1.88) | B DIC | 208120.8 |
| **NFHS 5 rudimentary housing** | **Constant** | -4.56 | 0.23 | 3708 | (-5.01, -4.11) | dbar | 199633.5 |
|  | **State** | 1.63 | 0.46 | 2693 | (0.95, 2.74) | thetabar | 187159.2 |
|  | **District** | 1.27 | 0.08 | 1592 | (1.12, 1.44) | effective no. of pars | 12474.3 |
|  | **Cluster** | 2 | 0.04 | 260 | (1.92, 2.08) | B DIC | 212107.8 |

**Supplementary Table 6:** Pearson correlation value (p-value) between Markov Chain Monte Carlo estimates and crude estimates for the district-level prevalence of three indicators of housing quality – finished housing, semi-finished housing, and rudimentary housing

|  | **Crude finished** | **Crude semi-finished** | **Crude rudimentary** |
| --- | --- | --- | --- |
| **MCMC Finished** | 0.99 (p<0.001) | - | - |
| **MCMC Semi-finished** | - | 0.99 (p<0.001) | - |
| **MCMC Rudimentary** | - | - | 0.99 (p<0.001) |
|  | **2021** | | |
|  | **Crude finished** | **Crude semi-finished** | **Crude rudimentary** |
| **MCMC Finished** | 0.99 (p<0.001) | - | - |
| **MCMC Semi-finished** | - | 0.99 (p<0.001) | - |
| **MCMC Rudimentary** | - | - | 0.99 (p<0.001) |

**Supplementary Table 7:** Percentage of households by wealth quintile, caste, education (of household head), and caste living in rudimentary, semi-finished, and finished housing in 2016 and 2021

|  | **Rudimentary housing** | | **Semi-finished housing** | | **Finished housing** | |
| --- | --- | --- | --- | --- | --- | --- |
|  | **2016** | **2021** | **2016** | **2021** | **2016** | **2021** |
| Poorest | 22% | 19% | 74% | 73% | 3% | 8% |
| Poorer | 8% | 7% | 67% | 56% | 25% | 37% |
| Middle | 2% | 2% | 40% | 30% | 57% | 68% |
| Richer | 0% | 0% | 18% | 15% | 81% | 85% |
| Richest | 0% | 0% | 9% | 10% | 91% | 90% |
| Scheduled Caste | 9% | 7% | 46% | 42% | 45% | 51% |
| Scheduled Tribe | 12% | 13% | 60% | 55% | 28% | 32% |
| Other backwards caste | 6% | 5% | 40% | 37% | 54% | 59% |
| None of the above | 4% | 3% | 31% | 27% | 64% | 70% |
| Don't know | 9% | 7% | 42% | 39% | 49% | 54% |
| No education | 11% | 10% | 55% | 51% | 33% | 40% |
| Up to 5th grade | 8% | 7% | 49% | 44% | 43% | 49% |
| Up to 8th grade | 6% | 6% | 43% | 40% | 51% | 54% |
| Up to 12th grade | 4% | 4% | 34% | 32% | 62% | 64% |
| Above 12th grade | 2% | 2% | 20% | 19% | 78% | 79% |
| Hindu | 7% | 6% | 43% | 39% | 50% | 55% |
| Muslim | 5% | 4% | 40% | 38% | 54% | 58% |
| Christian | 9% | 10% | 51% | 49% | 40% | 41% |
| Sikh | 1% | 2% | 34% | 34% | 66% | 64% |
| Other | 15% | 17% | 50% | 45% | 35% | 38% |

**Supplementary figure 1:** Rural India maps depicting the district-level prevalence of three indicators of housing quality in 2016. A. Finished housing B. Semi-finished housing C. Rudimentary housing. Decile cutoff values are based on the prevalence of each outcome in 2016.


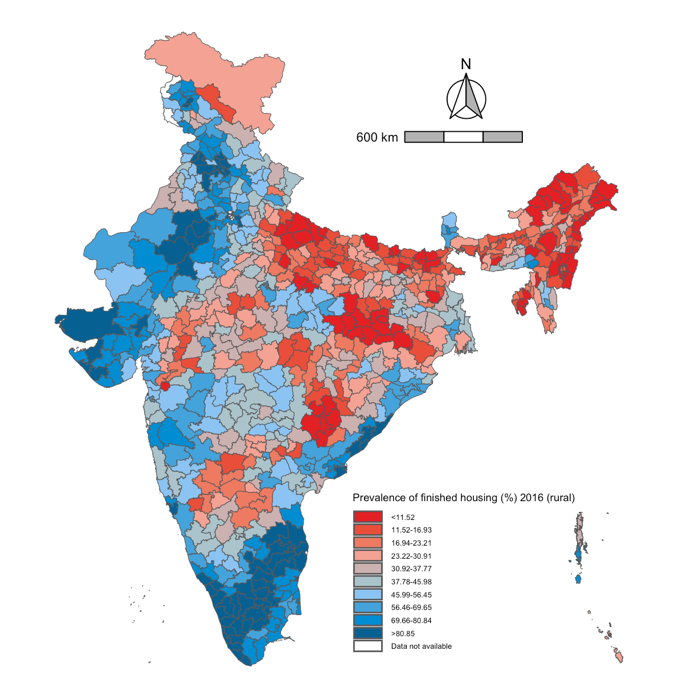

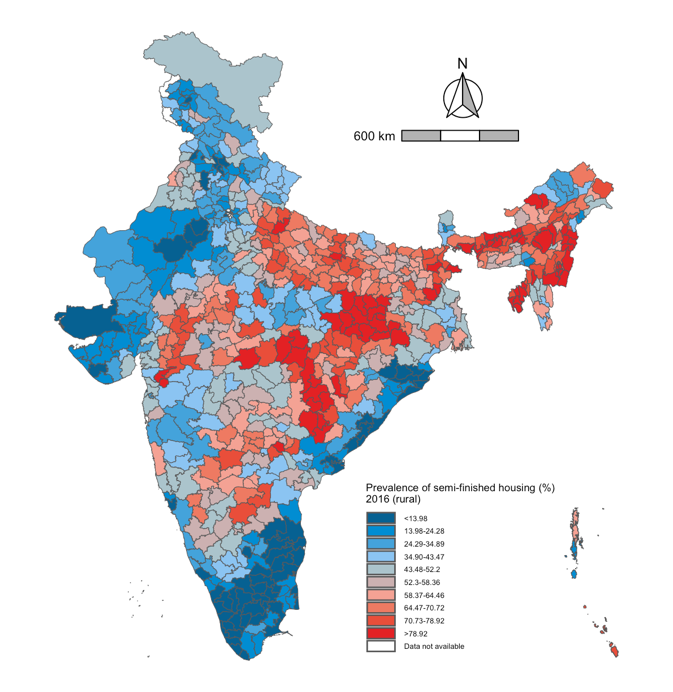
 **
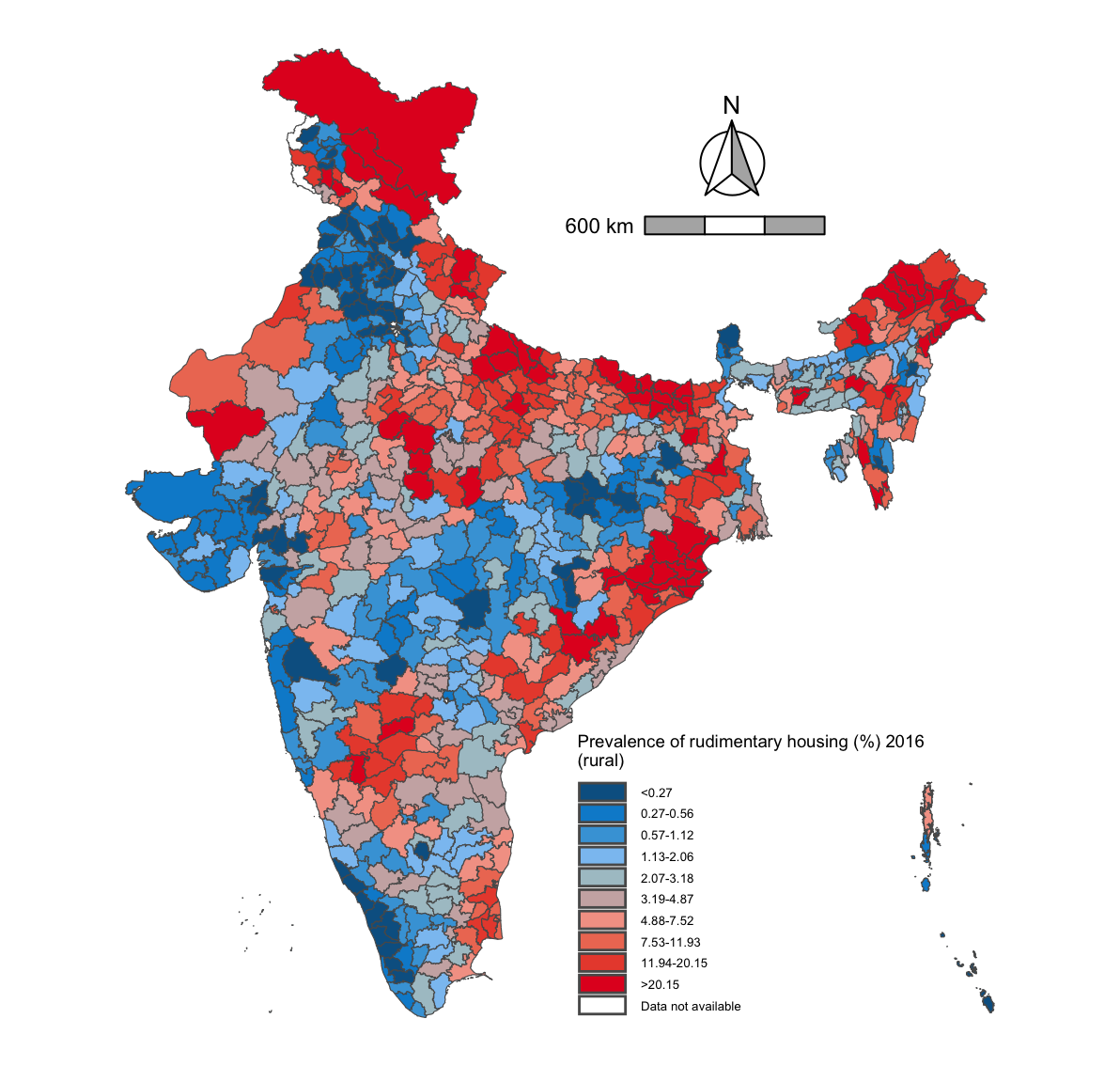
**

1. (B) (C)

**Supplementary figure 2:** Urban India maps depicting the district-level prevalence of three indicators of housing quality in 2016. A. Finished housing B. Semi-finished housing C. Rudimentary housing. Decile cutoff values are based on the prevalence of each outcome in 2016.


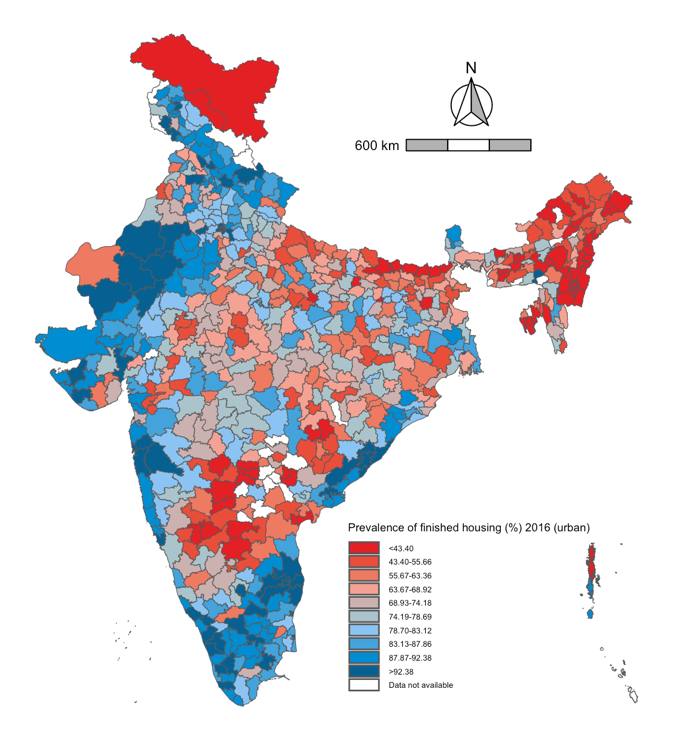

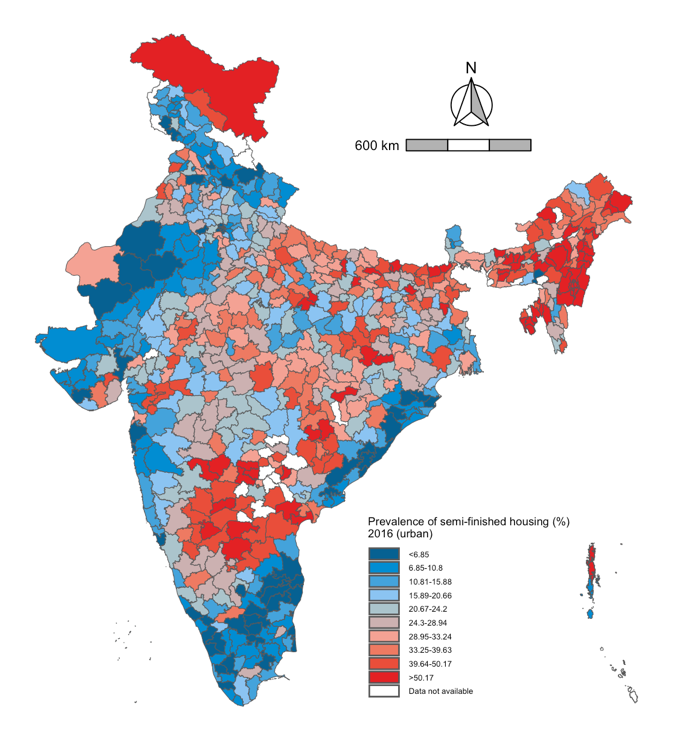

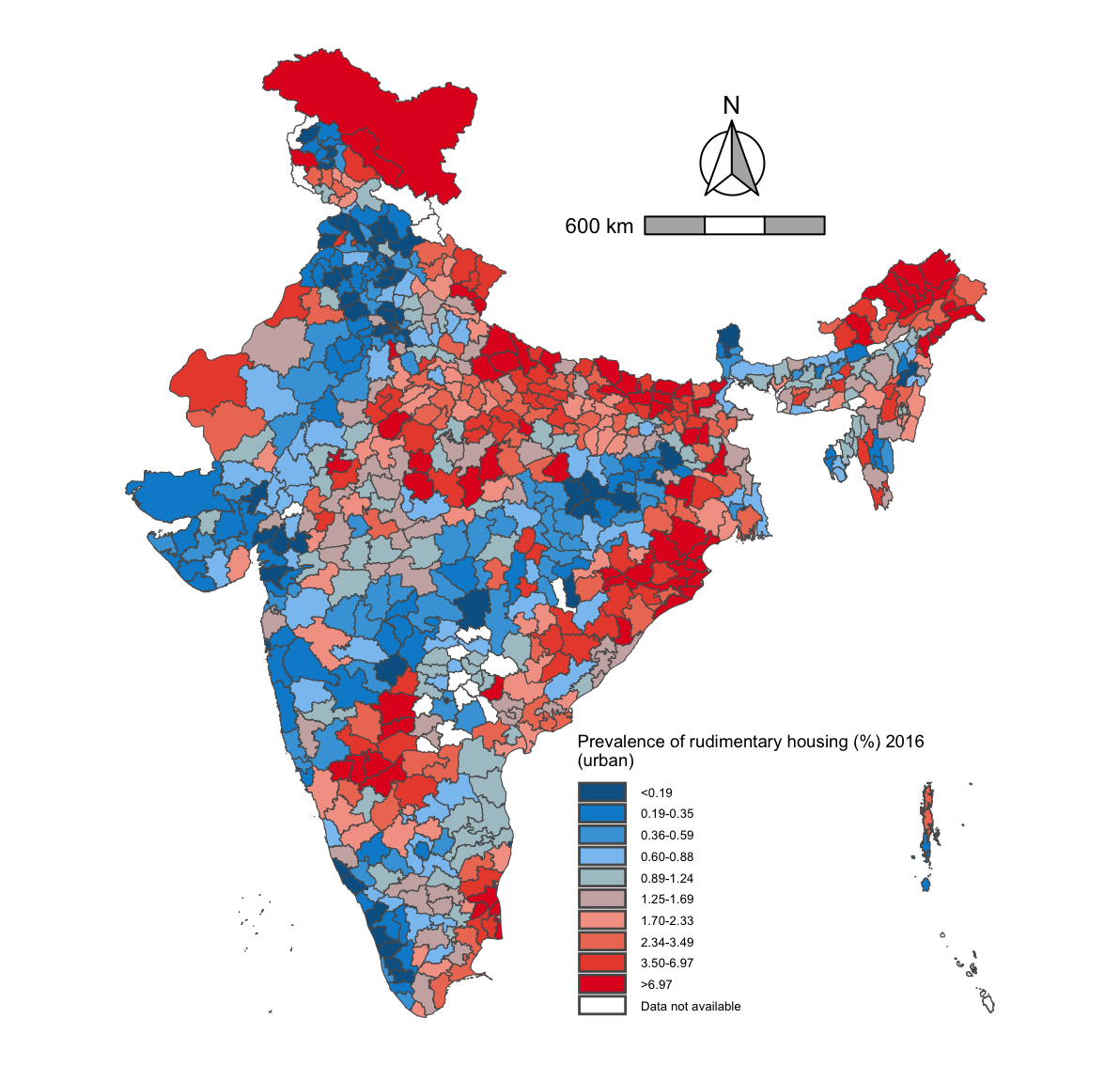


1. (B) (C)
